# Urine Microscopy Finding in a Patient with Cirrhosis and AKI

**DOI:** 10.34067/KID.0000000000000312

**Published:** 2024-02-29

**Authors:** Sheheryar Khan, Abraham W. Aron

**Affiliations:** 1 Highlands ARH Regional Medical Center, Hazard, Kentucky; 2 Georgetown University School of Medicine, Washington, DC

**Keywords:** AKI, clinical nephrology, liver failure

## Abstract

**Figure:**
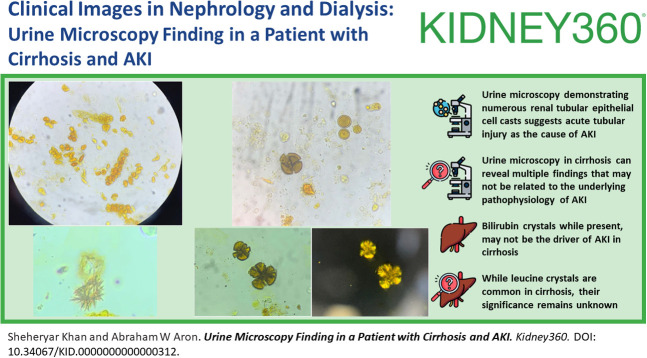

## Case Description

A 54-year-old woman with cirrhosis secondary to nonalcoholic steatohepatitis presented with hematemesis and abdominal pain in the setting of multiple bleeding varices. Initial laboratory workup was notable for potassium 6.6 mEq/L, serum creatinine 1.5 mg/dl, total bilirubin 4.7 mg/dl, and hemoglobin 8.8 g/dl. Her course was complicated by progressive hypotension and AKI, for which nephrology was consulted.

**Figure 1:**
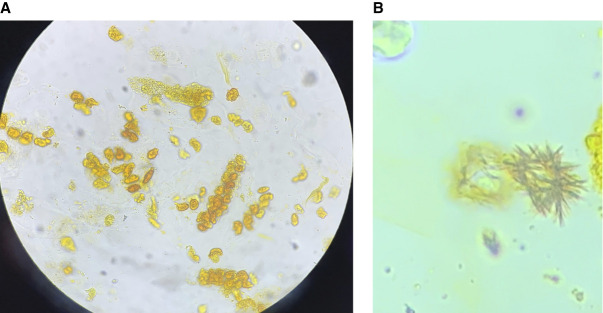
**Urine microscopy findings in cirrhosis.** (A) Bile-stained, renal tubular epithelial cell casts consistent with acute tubular injury. (B) Clumped, needle-shaped crystals consistent with bilirubin crystals.

**Figure 2:**
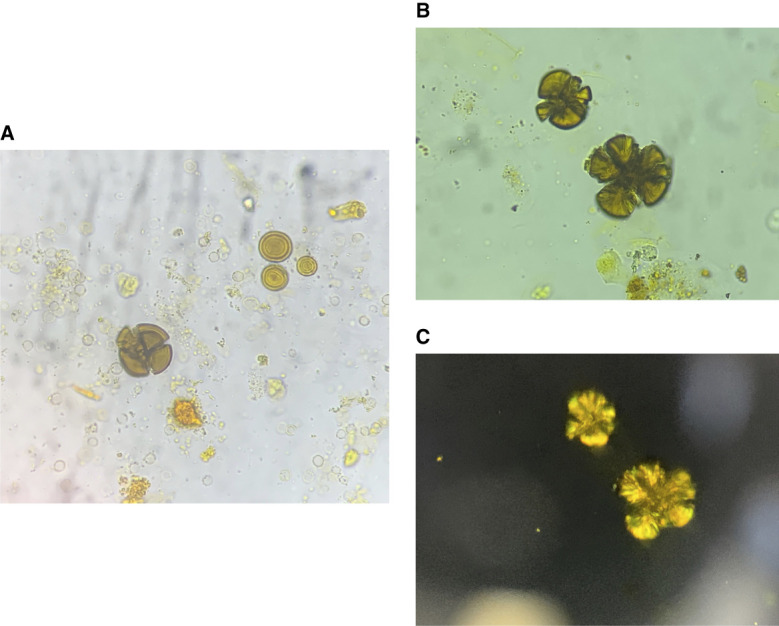
**Leucine crystalluria.** (A) Leucine crystals are brown, circular, laminated and can appear like an oil drop. (B) Fractured leucine crystals. (C) Leucine crystals strongly birefringent under polarized light.

Urine microscopy demonstrated bile-stained, renal tubular epithelial cell casts (Figure 1A); clumped, needle-shaped crystals; (Figure 1B) and brown, circular, laminated, oil drop crystals (Figure 2A) that were strongly birefringent under polarized light (Figure 2, B and C). The diagnosis of acute tubular injury with bilirubin and leucine crystals was made. Despite supportive care, her kidney function continued to deteriorate, and she was initiated on renal replacement therapy.

## Discussion

The significant physiologic derangements that occur in cirrhosis leads to a broad differential for AKI in this population, with prerenal azotemia (15%–45%), acute tubular injury (15%–60%), and hepatorenal syndrome type 1 (10%–40%) being the most common.^1^ Although bile acids have cytotoxic properties in vitro, bile cast nephropathy as an etiology for AKI is still hotly debated.^2^ In an autopsy study of patients with cirrhosis, 85% with hepatorenal syndrome were found to have tubular bile casts, proving that bile acid casts and subsequent crystalluria are common. Bilirubin crystals are typically yellow to green, clumped, needle-like crystals that are birefringent under polarized light.

Leucine is an essential, branched chain amino acid (BCAA). BCAAs (including leucine, isoleucine, and valine) have been shown to promote anabolism and may help reduce cachexia. In cirrhosis, there is a reduction in BCAAs compared with aromatic amino acids, which is believed to contribute to the genesis of hepatic encephalopathy.^3^ While the first enzyme involved in BCAA metabolism is found primarily in the muscle, activity for the second enzyme complex involved, branched chain *α*-keto acid dehydrogenase, is the highest in the liver.^3^ Despite the potential for reduced branched chain *α*-keto acid dehydrogenase activity in cirrhosis, there does not appear to be increased plasma leucine levels in patients with cirrhosis after undergoing a BCAA load compared with healthy controls.^4^ It is unknown whether deranged BCAA metabolism in cirrhosis may chemically alter leucine or its metabolites to promote crystallization in the urine.

Leucine crystals are typically yellow to brown, circular crystals that are oily in appearance with concentric rings or laminations that may appear cracked. In addition, they are strongly birefringent under polarized light and may have a pseudo-Maltese cross appearance.^5^ Although the presence of leucine crystals is common in cirrhosis, their clinical significance and etiology remains elusive.

The urine microscopy from our patient, although not novel, demonstrates many of the classic urine microscopy findings associated with cirrhosis.

## Teaching Points

- Urine microscopy demonstrating numerous renal tubular epithelial cell casts suggests acute tubular injury as the cause of AKI.
- Urine microscopy in cirrhosis can reveal multiple findings that may not be related to the underlying pathophysiology of AKI.
- Bilirubin crystals, while present, may not be the driver of AKI in cirrhosis.
- While leucine crystals are common in cirrhosis, their significance remains unknown.
